# Ethical considerations and patient safety concerns for cancelling non-urgent surgeries during the COVID-19 pandemic: a review

**DOI:** 10.1186/s13037-021-00293-7

**Published:** 2021-04-29

**Authors:** Nolan J. Brown, Bayard Wilson, Stephen Szabadi, Cameron Quon, Vera Ong, Alexander Himstead, Nathan A. Shlobin, Chen Yi Yang, Brian V. Lien, Shane Shahrestani, Katelynn Tran, Ali R. Tafreshi, Jack Birkenbeuel, Seth C. Ransom, Elliot H. Choi, Ronald Sahyouni, Alvin Y. Chan, Aaron Kheriaty, Isaac Yang

**Affiliations:** 1 Department of Neurological Surgery, University of California, Irvine, Orange, CA USA; 2 Department of Neurological Surgery, University of California, Los Angeles, CA USA; 3 Western Michigan University Homer Stryker M.D. School of Medicine, Kalamazoo, MI USA; 4 John A. Burns School of Medicine, University of Hawaii at Manoa, Honolulu, HI USA; 5 Department of Neurological Surgery, Northwestern University Feinberg School of Medicine, Chicago, IL USA; 6 Keck School of Medicine of USC, Los Angeles, CA USA; 7 Department of Neurological Surgery, Geisinger Commonwealth School of Medicine, Danville, PA USA; 8 College of Medicine, University of Arkansas for Medical Sciences, Little Rock, AR USA; 9 Department of Neurological Surgery, University of California, San Diego, San Diego, CA USA; 10 Department of Psychiatry and Human Behavior, University of California, Irvine, CA USA; 11 University of California, Los Angeles, CA USA; 12 Departments of Neurosurgery, Los Angeles, CA USA; 13 Departments of Departments of Head and Neck Surgery, Los Angeles, CA USA; 14 Departments of Radiation Oncology, Los Angeles, CA USA; 15 Jonsson Comprehensive Cancer Center, Los Angeles, CA USA; 16 Los Angeles Biomedical Research Institute, Los Angeles, CA USA; 17 Harbor-UCLA Medical Center, Los Angeles, CA USA; 18 David Geffen School of Medicine, University of California Los Angeles (UCLA), Los Angeles, CA USA

**Keywords:** COVID-19, Non-urgent surgery, Ethics

## Abstract

At the time of writing of this article, there have been over 110 million cases and 2.4 million deaths worldwide since the start of the Coronavirus Disease 2019 (COVID-19) pandemic, postponing millions of non-urgent surgeries. Existing literature explores the complexities of rationing medical care. However, implications of non-urgent surgery postponement during the COVID-19 pandemic have not yet been analyzed within the context of the four pillars of medical ethics. The objective of this review is to discuss the ethics of elective surgery cancellation during the COVID-19 pandemic in relation to beneficence, non-maleficence, justice, and autonomy. This review hypothesizes that a more equitable decision-making algorithm can be formulated by analyzing the ethical dilemmas of elective surgical care during the pandemic through the lens of these four pillars. This paper’s analysis shows that non-urgent surgeries treat conditions that can become urgent if left untreated. Postponement of these surgeries can cause cumulative harm downstream. An improved algorithm can address these issues of beneficence by weighing local pandemic stressors within predictive algorithms to appropriately increase surgeries. Additionally, the potential harms of performing non-urgent surgeries extend beyond the patient. Non-maleficence is maintained through using enhanced screening protocols and modifying surgical techniques to reduce risks to patients and clinicians. This model proposes a system to transfer patients from areas of high to low burden, addressing the challenge of justice by considering facility burden rather than value judgments concerning the nature of a particular surgery, such as cosmetic surgeries. Autonomy can be respected by giving patients the option to cancel or postpone non-urgent surgeries. However, in the context of limited resources in a global pandemic, autonomy is not absolute. Non-urgent surgeries can ethically be postponed in opposition to the patient’s preference. The proposed algorithm attempts to uphold the four principles of medical ethics in rationing non-urgent surgical care by building upon existing decision models, using additional measures of resource burden and surgical safety to increase health care access and decrease long-term harm as much as possible. The next global health crisis will undoubtedly present its own unique challenges. This model may serve as a comprehensive starting point in determining future guidelines for non-urgent surgical care.

## Introduction

Coronavirus Disease 2019 (COVID-19) is caused by infection from the novel coronavirus SARS-CoV-2, a virus which first rose in Wuhan, China, and has – at the time of writing – led to over 110 million cases and 2.4 million deaths globally [1]. In response to the COVID-19 global pandemic, hospitals around the world were forced to postpone or cancel elective surgeries to combat the rapid spread of the virus [1]. The precise impact of that decision is still unclear but is likely to be dramatic. One report estimated that over a 12-week period of peak spread, as many as 28 million surgeries – approximately 70% of all elective surgeries worldwide – would be cancelled due to the pandemic [2]. In the United States, this translated to 343,670 cancellations per week [2]. Despite a concerted effort to restart elective surgeries at many institutions across the U.S. this summer, the resurgence of the virus this winter is likely to force many hospitals to consider canceling elective surgeries once more.

There is no shortage of literature examining the complexities of rationing medical care. Existing studies have discussed guidelines for resuming non-urgent elective surgeries [3–5], ethical considerations in rationing lifesaving resources [6–10], protocols and clinical decision flowcharts for reducing non-urgent elective surgery risk [5, 11–14], strategies to increase surge capacity [15], and predictive modeling for healthcare resource usage [2, 16, 17]. Current work related to the COVID-19 pandemic is sure to shed new light on many of these issues. This article addresses the ethics surrounding performance and cancellation of elective surgeries during the COVID-19 pandemic and discusses the difficulties of devising appropriate surgical triage algorithms in the face of waxing and waning threats of infection from COVID-19. This discussion is grounded in the four pillars of medical ethics – beneficence, non-maleficence, justice, and autonomy – as they relate to unique considerations brought about by the pandemic’s effects on elective (i.e., non-urgent) surgical care. Lastly, this paper proposes a triage algorithm for addressing elective surgical care during a widespread pandemic which aims to respect each principle to the extent possible.

## The four pillars of medical ethics

### Beneficence

During the first wave of the COVID-19 pandemic in the United States, many physicians and hospitals followed the Surgeon General’s guidelines to postpone non-essential medical, surgical, and dental procedures [12] in order to add to the supply of healthcare workers available to combat the pandemic and mitigate depletion of PPE (personal protective equipment) [13]. Postponements relied on a classification system which organized procedures into several categories based largely on urgency [18] (Tables 1 and 2). In this system, surgeries deemed less urgent (Tier 1a) were more readily cancelled or postponed, while other surgeries (Tier 2a or 2b) were addressed on a case-by-case basis. During the initial surge, the beneficence of cancelling non-urgent (i.e. lower tier) cases was clear; elective cancellations allowed for the reallocation of healthcare staff, space, and equipment towards the effort to fight the pandemic.

**Table 1:** Surgical Urgency Classification System

Tier	Definition	Action
1a	Low acuity surgery/healthy patient	Cancellation or postponement to reallocate resources and attention towards addressing the pandemic
1b	Low acuity surgery/unhealthy patient	Postpone/cancel or perform at ambulatory surgery center
2a	Intermediate acuity surgery/healthy patient	Postpone surgery on a case by case basis; consider ambulatory surgery center
2b	Intermediate acuity surgery/unhealthy patient	Postpone surgery on a case by case basis; consider ambulatory surgery center
3a	High acuity surgery/healthy patient	Do not postpone
3b	High acuity surgery/unhealthy patient	Do not postpone

**Table 2:** Examples of surgical case types stratified by indication and urgency

Indication	Urgency	Case Examples
Emergent	< 1 h	• Life-threatening emergencies • Acute exsanguination/hemorrhagic shock • Trauma level 1 activations • Acute vascular injury or occlusion • Aortic dissection • Emergency C-section • Acute compartment syndrome • Necrotizing fasciitis • Peritonitis • Bowel obstruction/perforation
Urgent	< 24 h	• Appendicitis/cholecystitis • Septic arthritis • Open fractures • Bleeding pelvic fractures • Femur shaft fractures & hip fractures • Acute nerve injuries/spinal cord injuries • Surgical infections
Urgent-elective	< 2 weeks	• Cardiothoracic/cardiovascular procedures • Cerebral aneurysm repair • Vascular access devices • Skin grafts/flaps/wound closures • Scheduled C-section • Closed fractures • Spinal fractures & acetabular fractures
Elective (essential)	1–3 months	• Cancer surgery & biopsies • Subacute cardiac valve procedures • Hernia repair • Hysterectomy • Reconstructive surgery
Elective (discretionary)	> 3 months	• Cosmetic surgery • Bariatric surgery • Joint replacement • Sports surgery • Vasectomy/tubal ligation • Infertility procedures

Examples of surgical case types stratified by indication and urgency. From: Stahel PF. How to risk-stratify elective surgery during the COVID-19 pandemic?. *Patient Saf Surg*. 2020;14:8. Published 2020 Mar 31. doi:10.1186/s13037-020-00235-9

Example of surgical urgency classification system. From: COVID-19: Guidance for Triage of Non-Emergent Surgical Procedures. https://www.facs.org/covid-19/clinical-guidance/triage.

The ethics of such a decision can seem straightforward in the short term; but the matter becomes complicated when considering the downstream effects of prolonged postponement, even for non-lethal conditions [2]. Delaying elective cases can lead to significant morbidity [2]. According to one study published early on in the pandemic, approximately 50% of cases meet criteria for being elective (i.e. non-emergent) and time-sensitive [12], and it is patients awaiting these surgeries who are at the highest risk of morbidity during postponements and cancellations [2]. A cancer patient – as an example – risks a fundamental change in prognosis if his or her surgery is postponed for any significant period of time.

Where exactly these patients fit in amongst other patients awaiting surgeries scheduled prior to the pandemic is difficult to determine, particularly considering the backlog of cases generated by any broad postponement. One predictive model formulated at the beginning of the pandemic estimated that it would take a median of 45 weeks to clear the global backlog of elective surgical cases resulting from COVID-19 delays, even assuming an optimistic 20% increase in case turnover post-pandemic [2]. When considering the morbidity suffered by patients with non-urgent – but time-sensitive – surgical pathology, prioritizing patients with COVID-19 at their expense may not, in fact, provide the greatest net benefit for the largest number of patients.

**Fig. 1:**
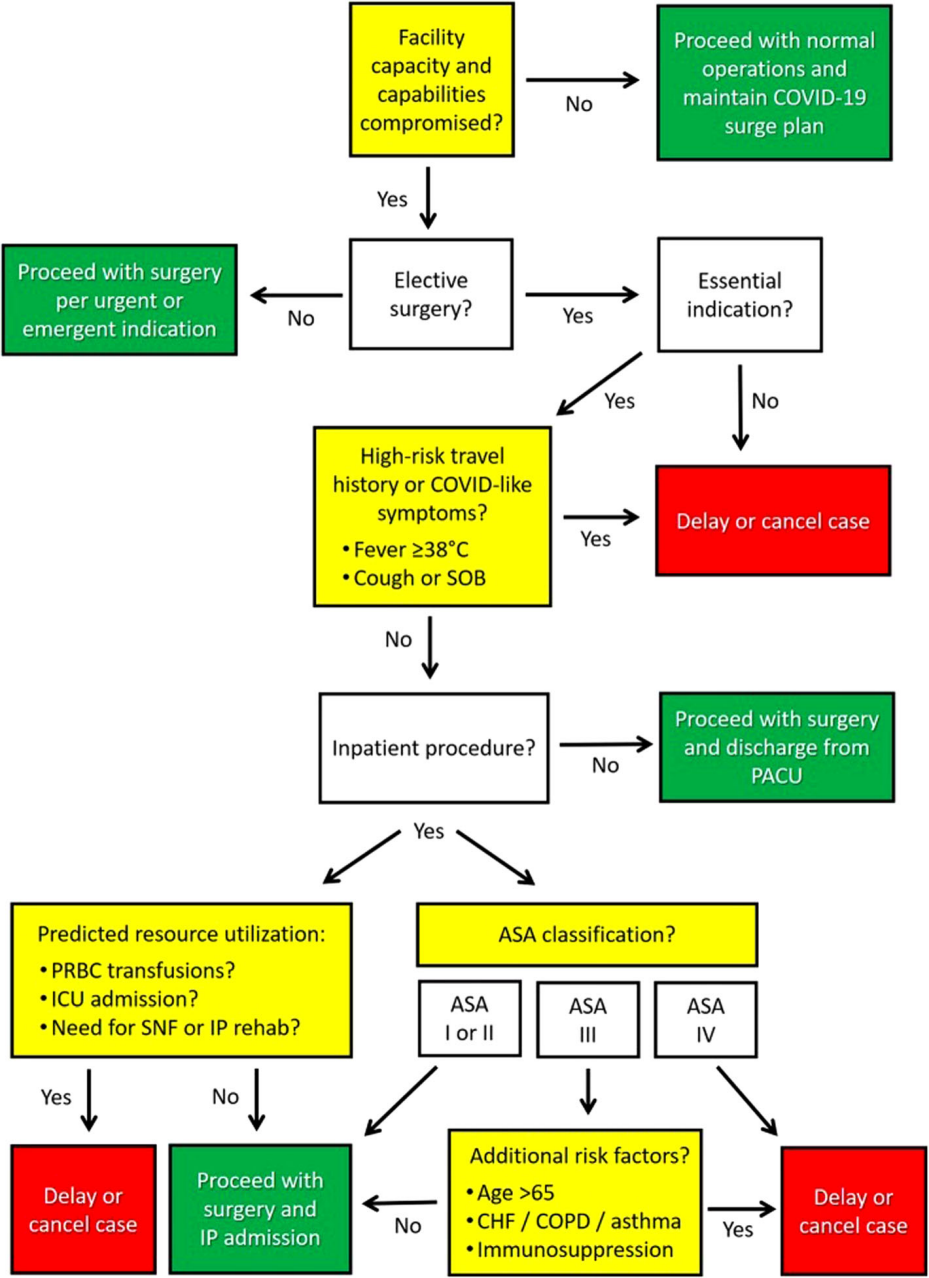
Proposed triage algorithm for COVID-19. From: Stahel PF. How to risk-stratify elective surgery during the COVID-19 pandemic?. *Patient Saf Surg*. 2020;14:8. Published 2020 Mar 31. doi:10.1186/s13037-020-00235-9

Pandemic related decisions around non-urgent elective surgeries involve weighing complicated long-term risks to public health. During a pandemic, the public health risk for a given community relates to several factors, including local healthcare resource strain, the rate of infection, the pattern of disease transmission, and the specific needs of regional patient populations. Triage schemes which have emerged as a result of the COVID-19 pandemic have tended to focus primarily on two of these factors – patient-specific COVID-19 infection risk and surgical severity – to arrive at a go/no-go decision. One example of such a triage scheme – proposed by Stahel et al. [12] – is shown in Fig. 1. In addition to factors such as high-risk travel and ASA (American Society of Anesthesiologists) grading, this triage algorithm incorporates elective surgical indications as well as the potential need for perioperative resources (blood transfusion products), requirement for extended ventilation and/or postoperative ICU (intensive care unit) admission, and an estimated hospital length of stay in order to determine whether or not a case should be delayed or cancelled.

To better adhere to the principle of beneficence, triage algorithms like that proposed by Stahel et al. could be augmented to allow for scaling of surgical delivery up and down according to local pandemic stressors. Researchers at the University of Pennsylvania have developed a predictive algorithm called CHIME (COVID-19 Hospital Impact Model for Epidemics) [19] which uses several measures of population size, hospital resource availability, and community viral transmission to make short-term local predictions for healthcare strain [16]. Including a component of local resource strain – such as that provided by the CHIME algorithm – could help maximize beneficence by ensuring that elective cases be considered at institutions where strain on local resources is relatively low. Any pandemic-related strategy focused on the beneficence principle must consider addressing non-urgent surgeries which can be safely performed in a timely manner in parallel with efforts to combat the pandemic, to maximize the benefits to patient suffering from non-urgent – but time sensitive – diseases.

### Non-maleficence

Patients brought to the hospital are at risk of spreading and contracting disease. The principle of non-maleficence, often referred to as the “do no harm” principle, strives to minimize the risk of harm to a patient, and argues that any procedure whose anticipated harms outweigh the expected benefits should not be performed [20]. During the current pandemic, the decision to suspend non-urgent elective procedures was made in part to protect surgical patients from disease transmission (i.e., harm).

It should be noted, however, that the principle of non-maleficence can – and should – extend beyond the surgical patient [21]. Harm must be considered as it applies to healthcare workers, other patients in the hospital, and the public at large. One study published at the outset of the pandemic reported that 41% of COVID-19 infections in China were hospital acquired [22]. Harm also does not need to be direct to be severe; in the context of scarce resources, electing to ventilate a patient for a non-urgent surgery harms the patient who dies because a ventilator was not available. Moreover, direct harm suffered by healthcare workers tasked with staffing an operating room can be indirectly transferred to future patients whose care they cannot provide [8].

Institutional triage protocols – such as that proposed by Stahel (Fig. 1) – attempt to reduce harm to all groups involved by stratifying known risks. These include potential harm to the prospective patient by stratifying clinical urgency, as well as to other patients, healthcare workers, and the public health infrastructure as a whole by evaluating viral transmission risk and anticipated resource utilization. Most surgical triage protocols developed during this pandemic effectively minimize non-maleficence with respect to COVID; the extent to which they minimize non-maleficence more broadly is less clear. As stated previously, postponement of certain non-urgent surgeries can cause harm [2] to patients with time-sensitive pathologies. As such, a constant effort should be made to reintroduce non-urgent, time-sensitive surgeries to institutions which can support them. Ensuring that these cases are performed safely will require a more stringent set of procedures than those utilized prior to the pandemic. These precautionary measures should incorporate telehealth prescreening, regimented testing protocols before surgery, tailoring of surgical techniques to minimize production of aerosols, alternative anesthesia protocols to reduce healthcare worker exposure, and protocols to reduce post-operative coughing and nausea which may further viral spread [5, 11]. Each of these measures rests on the necessary assumption that every patient is potentially Sars-CoV-2 positive [5].

### Justice

In the state of California in late March 2020, protocols regarding the handling of non-urgent elective procedures varied considerably among medical centers [3, 23]. During this time, theoretically, a patient with a non-urgent surgical problem could have their surgery denied by one institution and granted by another in the same state. At face value, this does not appear to comply with the principle of justice, which demands equitable treatment across patient groups regardless of location.

To maintain justice in the formulation and implementation of triage protocols it is vital that decision-making criteria are transparent, responsive to the general will of the public [6], and based primarily on medical merit [7] as opposed to solely respecting conservation of resources [8]. Transparency is fundamental to ensure public trust in the healthcare system; it minimizes psychological harm to patients who may be denied elective surgery and reduces moral injury to physicians who might otherwise need to make hasty clinical decisions [7]. Triage algorithms are effective at stratifying patients into groups, but as resources become scarcer, the justice principle becomes harder to follow.

When COVID-19 surged in parts of the United States in late March of 2020, some institutions continued performing elective operations widely considered to be of low priority, such as cosmetic surgeries [3]. This decision might have seemed unjust to the casual observer, but an argument can be made that performing these surgeries during a pandemic respects the justice principle. Cosmetic surgeries, for example, are often outpatient surgeries (not requiring an inpatient bed), less complex, shorter in duration, and are usually performed on healthier patients [5]. For hospitals capable of managing COVID-19 transmission risk – or faced with relatively low levels of COVD-19 cases – delaying these forms of elective surgery could arguably violate the justice principle. Nevertheless, proponents of canceling cases cite a lack of immediate harm from postponement; in an interview with The Atlantic Magazine in March 2020, Dr. Gerard Doherty, Chair of Surgery at Brigham and Women’s Hospital, suggested that 25% of surgeries can be postponed without harm [24]. Merits of this argument notwithstanding, the issue of justice remains unaddressed. Considering the lack of clarity with respect to the pursuance of time-insensitive surgeries, a separate arm for these surgeries should be added to existing triage algorithms to address the issue of justice.

An intuitive suggestion to respect justice might be to implement and follow a uniform standard within a region or state, enabling all patients to be subject to a consistent set of rules and regulations. Realistically, though, adherence to justice in the face of a pandemic is more likely to manifest heterogeneously, since adhering to the same standard for elective surgeries across different hospitals can overstretch some hospitals more than others.

As such, the justice principle does not demand a blanket application of restrictions that unduly restrict certain patient groups from elective surgeries, even in cases of surgeries which are not time sensitive. Rather, it values emphasizing equal opportunity to receive elective surgeries where they may safely be performed.

### Autonomy

Patient autonomy is a crucial component of a successful physician-patient relationship. Although some hospital systems may enjoy redundancy of certain resources, others have experienced resource shortages which have had life-or-death implications for those suffering from COVID-19 [12]. The pandemic has altered many standard practices relating to patient autonomy, including the privacy of patient medical records and the principle of informed consent [7]. Since the pandemic began, for example, pre-surgical informed consent has required that patients be informed of the risk of exposure to COVID-19 [5]. Patients also forfeit the right to keep the results of their COVID tests private while in the hospital setting. While respecting patient autonomy is an important consideration when discussing the ethics of a particular policy, it is rarely sufficient to arrive at a decision to treat. It is, however, sufficient to arrive at a decision not to treat. The pandemic has provided ample opportunity to clarify this distinction, particularly given the evolving understanding of COVID-19 treatment. Not long into the first nationwide surge in the United States, physicians were placed in the precarious position of handling patient requests for therapies which were untested, ineffective or even unsafe [25]. In such circumstances, patient autonomy conflicted directly with non-maleficence, both for the individual patient in question and the broader healthcare community [7].

In this context, this paper argues that physicians can ethically postpone a non-urgent elective procedure even if postponement conflicts with a patient’s demands. Triage algorithms can make such a decision easier by removing bias from decision making, promoting a more ethical distribution of available surgical resources, and by providing a clear framework for evaluating when a patient’s right to autonomy does or does not trump the needs of public health.

It is important to mention that increasing patient autonomy can in some cases actually lead to decreased strain on a healthcare system [12]. Throughout the pandemic, patients have themselves volunteered to cancel or postpone various surgeries, which has reduced the immediate burden on hospitals and freed up personnel and resources to treat other patients [12]. While most triage algorithms do not explicitly mention a patient’s decision to pursue or forego surgery in its decision tree, it is nonetheless implied given the critical role of patient autonomy in any surgical decision making. In the context of this pandemic, patient autonomy should only supersede other considerations in cases where patients elect to forego surgery themselves.

## Conclusion

**Fig. 2:**
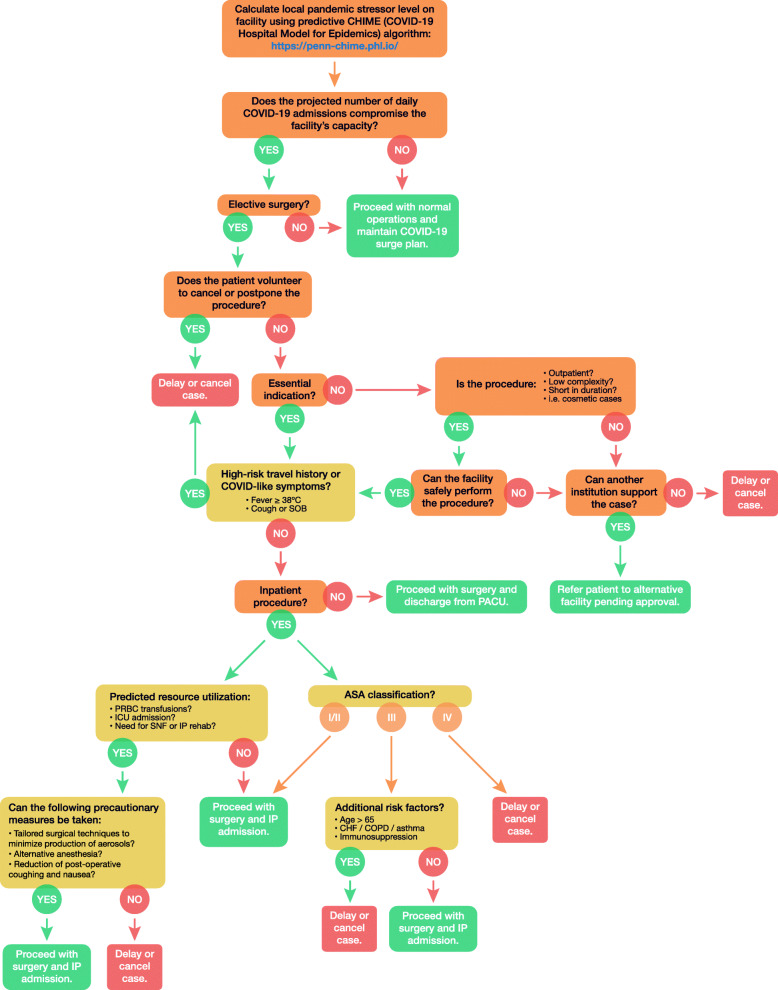
This review’s proposed COVID-19 Triage Algorithm built upon Stahel PF’s model using the ethical framework of beneficence, non-maleficence, justice, and autonomy

The ethics of postponing non-urgent elective surgical procedures during this COVID-19 pandemic are complex. Each tenet of medical ethics can be referenced to support or contest cancellations. Ultimately, while the decision to postpone elective cases was made appropriately in the short term during first surge of COVID-19 cases, it would be reasonable to expect downstream consequences which are harmful to patients whose surgeries were postponed. This paper’s best efforts to maximize beneficence and minimize non-maleficence during this pandemic have produced variations on a foundational triage algorithm for delivering surgical care (Figs. 1 and 2). No two hospitals face the exact same set of circumstances with respect to this pandemic, and so we can expect to see each institution navigate a return to elective surgeries differently. This patchwork approach to managing elective surgical cases is fraught with negative externalities, many of which are highlighted above, but as with any complex ethical problem, no solution exists without causing some harm.

Until 2020, much of the work done to optimize triage algorithms for pandemic preparedness relied on smaller scale outbreaks such as the 2009 H1N1 or 2014 Ebola epidemics. The scale and severity of this pandemic has forced local, regional, and national governments to work outside the bounds of existing algorithms, and forced most developed healthcare systems to temporarily postpone elective surgeries as a result. While the decision to postpone elective surgeries might not have been avoidable, we contend that postponing these surgeries in the most ethical manner should involve relying on a triage algorithm which maximizes beneficence and minimizes non-maleficence – such as that proposed by Stahel et al. – but also incorporates local variability in resources and disease burden to maximize justice. An example of this modified algorithm is shown in Fig. 2. In addition to including the same core decision tree as previous algorithms, this example addresses patient autonomy and the justice principle directly, and stresses the needed collaboration between healthcare providers and recipients. As is clear from the figure, this algorithm is complex, but its implementation necessitates considering factors such as hospital surge capacity, available ICU beds, PPE, ventilator availability, local COVID-19 disease burden, and rates of transmission. Nevertheless, more comprehensive algorithms enable a degree of flexibility which is less vulnerable to ethical criticism, and (hopefully) more effective in maximizing quality care to patients. As the United States prepares for another major surge, these considerations will be paramount to limit the delayed consequences of another widespread postponement of non-urgent elective cases.

## Data Availability

All data and materials support published claims and comply with field standards.
